# Evolution of Welding Residual Stresses within Cladding and Substrate during Electroslag Strip Cladding

**DOI:** 10.3390/ma13184126

**Published:** 2020-09-17

**Authors:** Mu Qin, Guangxu Cheng, Qing Li, Jianxiao Zhang

**Affiliations:** 1 School of Chemical Engineering and Technology, Xi’an Jiaotong University, Xi’an 710049, China; qinmu.1990.0113@stu.xjtu.edu.cn (M.Q.); liqingl@stu.xjtu.edu.cn (Q.L.); 2 Lanzhou LS Heavy Equipment CO., LTD, Lanzhou 730300, China; zhangjianxiao@lshec.com

**Keywords:** finite element model, electroslag strip cladding, welding, residual stress evolution

## Abstract

Hydrogenation reactors are important oil-refining equipment that operate in high-temperature and high-pressure hydrogen environments and are commonly composed of 2.25Cr–1Mo–0.25V steel. For a hydrogenation reactor with a plate-welding structure, the processes and effects of welding residual stress (WRS) are very complicated due to the complexity of the welding structure. These complex welding residual stress distributions affect the service life of the equipment. This study investigates the evolution of welding residual stress during weld-overlay cladding for hydrogenation reactors using the finite element method (FEM). A blind hole method is applied to verify the proposed model. Unlike the classical model, WRS distribution in a cladding/substrate system in this study was found to be divided into three regions: the cladding layer, the stress-affected layer (SAL), and the substrate in this study. The SAL is defined as region coupling affected by the stresses of the cladding layer and substrate at the same time. The evolution of residual stress in these three regions was thoroughly analyzed in three steps with respect to the plastic-strain state of the SAL. Residual stress was rapidly generated in Stage 1, reaching about −440 MPa compression stress in the SAL region at the end of this stage after 2.5 s. After cooling for 154 s, at the end of Stage 2, the WRS distribution was fundamentally shaped except for in the cladding layer. The interface between the cladding layer and substrate is the most heavily damaged region due to the severe stress gradient and drastic change in WRS during the welding process. The effects of substrate thickness and preheat temperature were evaluated. The final WRS in the cladding layer first increased with the increase in substrate thickness, and then started to decline when substrate thickness reached a large-enough value. WRS magnitudes in the substrate and SAL decreased with the increase in preheat temperature and substrate thickness. Compressive WRS in the cladding layer, on the other hand, increased with the increase in preheat temperature.

## 1. Introduction

As key equipment in petrochemical engineering, hydrogenation reactors play an important role in hydrodesulfurization, hydrocracking, and hydrofining processes [1,2]. During the fabrication of these high-hydrogen-pressure and high-temperature reactors, internal cladding made by austenitic stainless steel is required to increase corrosion resistance [3,4]. One such steel type, 2.25Cr–1Mo steel, is widely used for these reactors, and its properties at the evaluated temperatures levels have been investigated. However, with the development of hydrogenation technologies, higher operation pressures and temperatures have gradually led to replacement of the classical 2.25Cr–1Mo steel with V-reinforced Cr–Mo steel (2.25Cr–1Mo–0.25V). The addition of V allows the operation temperature to be increased up to 500 °C [1], but also results in a new problem. V increases reheat–cracking susceptibility during intermediate–stress–relief (ISR) treatment [5]. Moreover, welding residual stress (WRS) is relevant to the generation of reheat cracking. Nevertheless, the internal surface cannot withstand the corrosion environment without a protective cladding layer.

For large-scale internal cladding, electroslag strip cladding (ESSC) and submerged arc strip cladding (SASC) are preferentially applied using welding because of their low deposition rates, stable quality, and smooth welding surface [6,7,8,9]. For a hydrogenation reactor, the internal weld overlay is typically double-layered or multilayered consisting of intermediate and surface layers. To satisfy some specialized requirements, a new technology with a single-layer austenitic stainless steel of type 347L welded by ESSC was developed [10].

Complex WRS introduced in the ESSC process severely affects the performance of the welded structure, especially regarding toughness, fatigue life, and reheat cracking [1,11]. It is very difficult to measure and simulate WRS. In recent years, many methods have been used to measure residual stress, but their accuracy has not been sufficient. In addition, some simulations have been developed to analyze the final distribution of residual stress [12,13,14,15,16,17,18,19,20].

In spite of the abundance of studies on WRS in recent decades, reports on the evolution of WRS during cooling are rare. Makoto Udagawa et al. [9,21,22,23,24,25] proposed a 3D model to evaluate final WRS and the effect of post-weld heat treatment (PWHT). In [9], numerical simulations were performed on the basis of thermal elastic–plastic–creep analysis to evaluate WRS distribution produced from welding and PWHT. The effects of WRS on the stress-intensity factor (SIF) for various crack sizes were evaluated under typical pressurized-thermal-shock conditions. Results showed that tensile residual stress of 400 MPa occurred in the cladding layer at room temperature after PWHT, and the SIF was affected by the WRS of weld-overlay cladding and PWHT. In addition, the welding method was used to analyze the effects of WRS on the structural integrity of reactor pressure vessels [21]. Deep-hole-drilling (DHD) methods were used to evaluate WRS distribution through the weld fusion line [23,24,25]. The results of WRS simulation show reasonable agreement with the experiment results. The main cause of WRS due to welding and PWHT was the difference in thermal expansion between weld and base metals.

R. Lostado-Lorza [26,27,28] applied plastic-strain-range memorization on the basis of time-independent cyclic-plasticity theory for welding structures with single V-groove finite-element (FE) models that were manufactured by gas metal arc welding (GMAW). The theory combined the isotropic-hardening and non-linear kinematic-hardening rules (Chaboche model) to reproduce the behavior of cyclic plasticity and, thus, obtain residual stresses using welded-joint FE models. The authors also introduced an optimized welding parameter for FE modeling of the thermomechanical behavior of a V-groove welding-structure that was manufactured by GMAW. These described works are significantly important for studies establishing a numerical model of WRS, and have served as a valuable reference, providing values for numerical-simulation parameters used in the subsequent described research.

Y. Javadi et al. [11] introduced a contour-mapping method to measure complex structures, combined with sequential weld-buttering and -cladding operations, and compared the results with measurements made by incremental hole drilling. The results showed that both buttering and cladding introduce near-yield levels of tensile WRS, and the PWHT conducted after welding would almost relieve WRS. Prasad et al. [29] studied the microstructure and mechanical properties of ESSC and explosive cladding. The diffusion of elements was significant in ESSC; the shear strength, notch tensile strength, and impact toughness of explosive cladding were significantly greater than those in ESSC. Takuya Nagai and Shigetaka Okano [30,31] evaluated the influencing factors on WRS using X-ray stress measurements, and verified the results using the FEM. However, the evolution of residual stress was not studied. A better understanding of residual-stress evolution is very useful when studying the residual-stress distribution of complex structures.

The presence of WRS significantly affects the performance of welded structures, especially regarding toughness, fatigue life, and reheat cracking [32]. As mentioned above, the measurement method and FEM of WRS were significantly improved. Nonetheless, the evolution of WRS during cooling was not studied.

In this study, a semi-infinite axisymmetric two-dimensional (2D) cladding/substrate system using a finite-element model was established to capture primary WRS. The model was divided into three regions on the basis of significant differences in residual-stress distribution: cladding layer, stress-affected layer (SAL), and substrate. A blind hole method was applied to measure the WRS on the surface of test specimen. The evolution of residual stress in these three regions was analyzed in detail, especially for the SAL. Moreover, the evolution processes of WRS in these three regions were thoroughly analyzed in three steps with respect to the plastic strain state of the SAL. The effects of preheat temperature and substrate thickness were also evaluated.

## 2. Experimental Procedure

**Figure 1:**
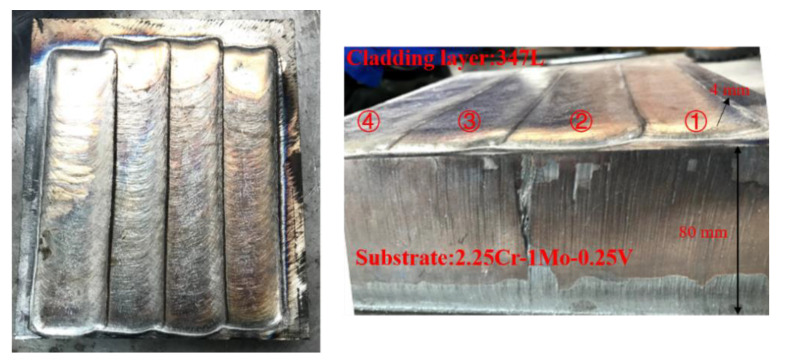
Electroslag strip cladding test plate.

**Figure 2:**
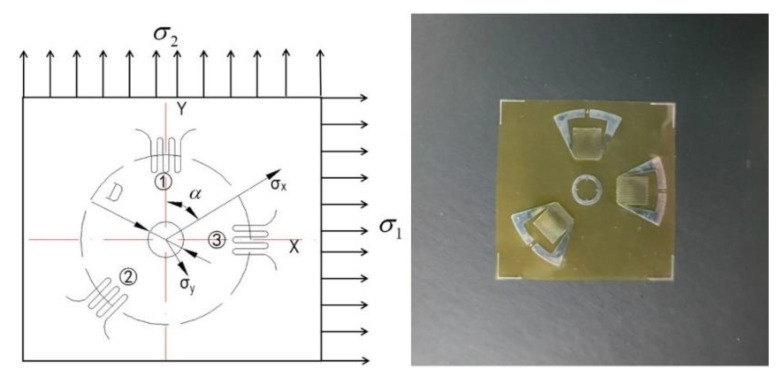
Diagram of the strain gauge.

**Figure 3:**
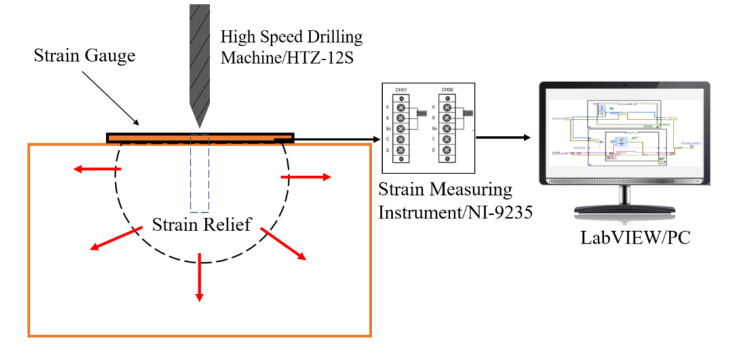
Experimental schematic diagram for measuring welding residual stress (WRS) of blind hole method.

The substrate material used in this work was provided by Arcelor Mittal (Steelmaker, Uchino, France). The specimen was cut from a large plate and processed into a square plate with a thickness of 80 mm and a length of 200 mm. Table 1 shows the chemical compositions and supply state of the base material. The normalizing and tempering temperature of supplied substrate material were 910 °C and 720 °C, respectively. The cladding layer was 347L stainless steel strip with about 2 mm thickness. The specimen was welded by the electroslag strip cladding method with 10SW-Sandvik as a flux in Lanzhou LS Heavy Equipment CO., LTD (Lanzhou, Gansu, China). After preheating the base material, 4 passes cladding layer were welded on the surface in sequence, as shown in Figure 1. The welding current and arc voltage were 775 A and 30.0 V, respectively. Welding speed was controlled at 120 cm/min. After completing all welding processes, a blind hole method was applied to measure the WRS on the upper and lower surfaces of the welded piece in this paper. In principle, for a residually stressed body, the stress will be released at the locations of the hole even for a very small diameter. This is attributed to the zero shear and normal stresses on the principal axis perpendicular to a free surface. The redistribution of stress in the surrounding region will cause the local strains on the surface to change correspondingly. A BHI120-3CD-(11) strain gauge was chosen to be the gauge that measure the variation of the local strain, as shown in Figure 2. The strain gauge in three different directions can measure three different locations’ strain $\varepsilon_{1}$, $\varepsilon_{2}$ and $\varepsilon_{3}$ respectively, which relate to the maximum and the minimum principal stress in the drilled hole. The maximum and the minimum principal stress were calculated using the following Equations (1)–(3). Constants *A* and *B* in Equations (1)–(3) are stress relief factors related to the type of strain gauges, drilling parameters and measured material properties. The measurement principle diagram is shown in Figure 3.

(1) $$\sigma_{x}=\frac{\varepsilon_{1}+\varepsilon_{3}}{4A}-\frac{1}{4B}\sqrt{{\left(\varepsilon_{3}-\varepsilon_{1}\right)}^{2}+{\left(\varepsilon_{3}+\varepsilon_{1}-2\varepsilon_{2}\right)}^{2}}$$

(2) $$\sigma_{y}=\frac{\varepsilon_{1}+\varepsilon_{3}}{4A}+\frac{1}{4B}\sqrt{{\left(\varepsilon_{3}-\varepsilon_{1}\right)}^{2}+{\left(\varepsilon_{3}+\varepsilon_{1}-2\varepsilon_{2}\right)}^{2}}$$

(3) $$\tan2\alpha =\frac{\varepsilon_{3}+\varepsilon_{1}-2\varepsilon_{2}}{\varepsilon_{1}-\varepsilon_{3}}$$

## 3. Model Formulation

Welding is a very complex process involving molten-metal, heat-transfer, metallurgical-reaction, mass-diffusion, solid-phase-transformation, and other processes [14,33]. If all these factors are considered in the model, WRS cannot be effectively simulated. In this study, some appropriate assumptions were made for effective simulation. For example, variations in the yield strength and volume induced by solid-phase transformation were not considered.

In this study, an uncoupled thermomechanical finite-element model was established using ABAQUS 6.13 software to simulate WRS distribution. First, temperature distribution was independently considered in thermal analysis. Then, the mechanical model was established by using geometric dimensions identical to those used in thermal analysis. The temperature-field results of thermal analysis were established as a predefined field in mechanical analysis. This means that the thermal-analysis results acted as a load in mechanical analysis. The boundary condition, meshed element type, and calculation step type were accordingly adjusted from thermal to mechanical analysis.

**Figure 4:**
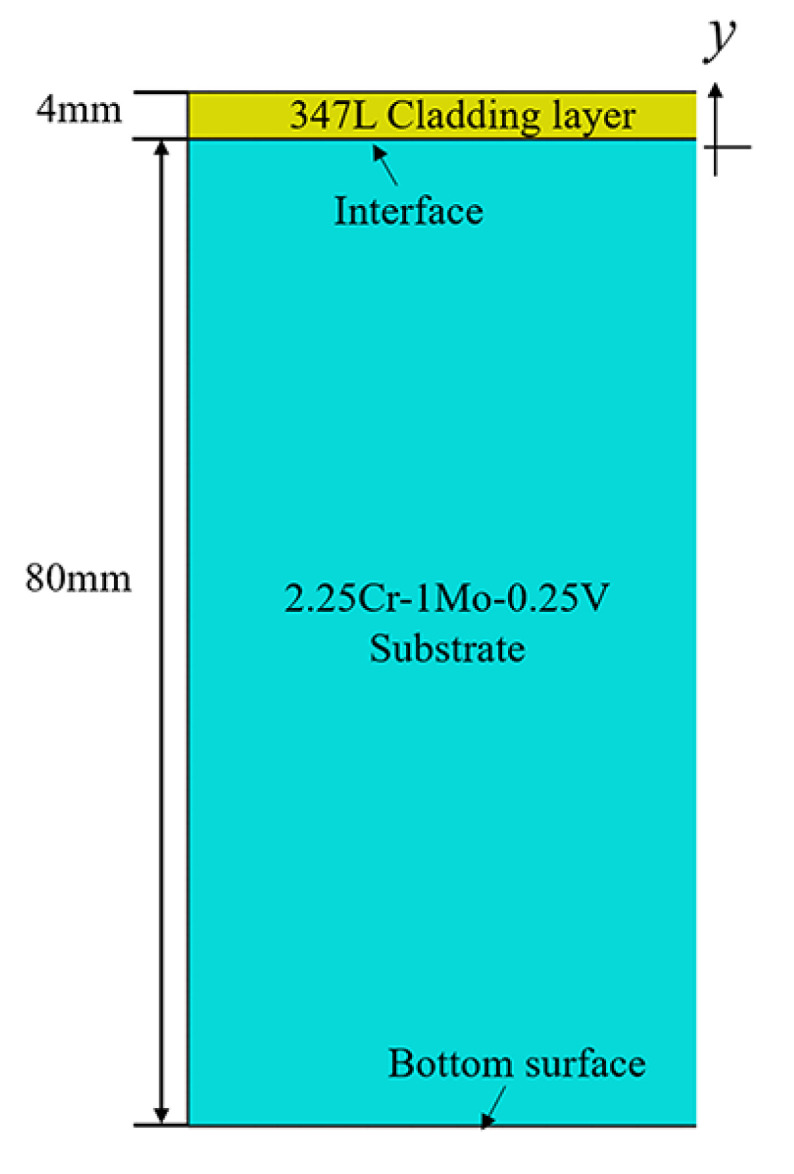
Geometric finite-element (FE) dimensions.

As mentioned above, the substrate of the system was a 2.25Cr–1Mo–0.25V steel with thickness of 80 mm. The thickness of cladding welded in a single layer is typically 4–4.5 mm thick in practice. Without loss of generality, 347 L austenitic stainless steel with 4 mm (*h*_CL_) cladding thickness was selected; the geometric FEM dimensions are shown in Figure 4. The cladding/substrate system consisted of 2.25Cr–1Mo–0.25V steel with 80 mm (*h*_Sub_) thickness as the substrate and 347 L steel as 4 mm thick cladding.

### 3.1. Thermal Analysis

**Figure 5:**
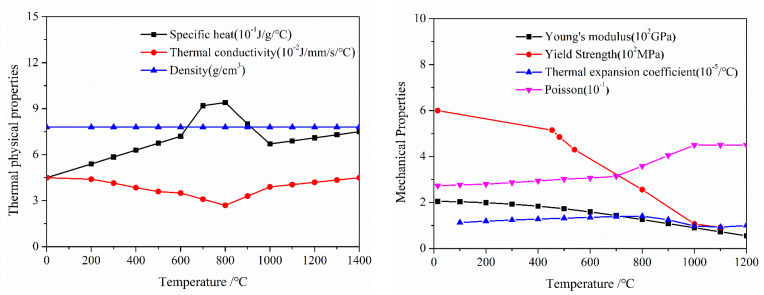
Temperature dependency of (**a**) thermal physical and (**b**) mechanical parameters of 2.25Cr–1Mo–0.25V.

**Figure 6:**
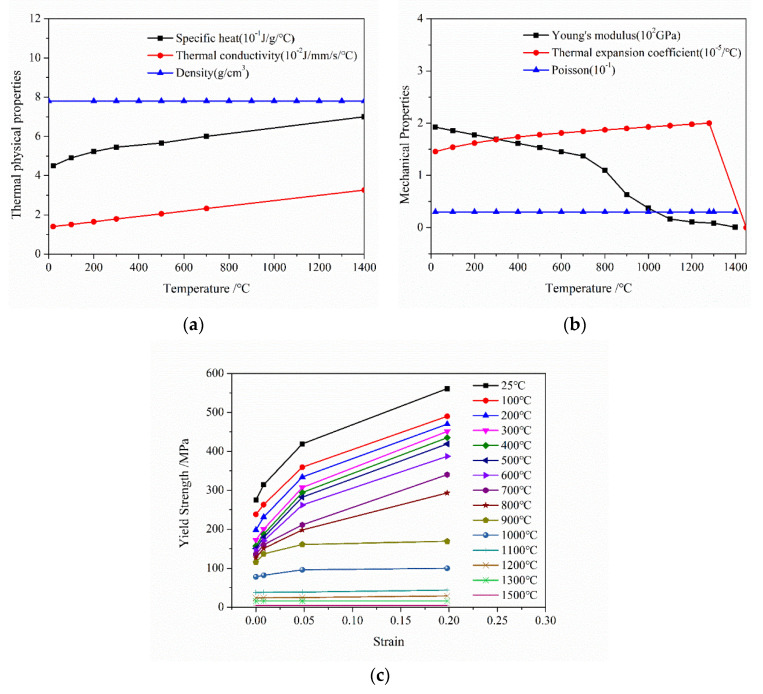
Temperature dependency of (**a**) thermal physical and (**b**) mechanical parameters, and (**c**) yield strength of 347 L steel.

In the thermal analysis step, temperature evolution in the cladding/substrate system was calculated in detail, especially for the cooling process which WRS was mainly generated. The temperature dependencies of the thermal physical and mechanical parameters of 2.25Cr–1Mo–0.25V substrate and 347 L cladding are shown in Figure 5 and Figure 6 [22], respectively. Figure 6 shows that the tensile stress of 347 L steel at temperature levels from 25 to 1500 °C was expressed and captures the strain change in the cladding layer to a great extent. To obtain the precise temperature distribution, the effects of convective heat transfer and heat radiation were considered both at the bottom and the top surfaces. The values for convective heat-transfer and heat-emissivity coefficients were 10 W·m^−2^·K^−1^ and 0.7, respectively [21]. The preheat temperature of the substrate was assumed to initially be 200 °C, and the effects of preheat temperature are discussed later in this paper.

In practice, the heat source in ESSC was close to the “wide width double-ellipsoid model”. In this study, however, the heat source was simplified as a predefined uniform temperature cladding metal with 2000 °C, because the model was 2D and the emphasis of this study was to analyze the evolution of WRS. A uniform temperature distribution heat source was applied in Song’s [33] research, and its simplicity is practical here. Next, the welded system started to cool as heat was transferred to the substrate and environment. The cooling time was set at 4000 s, enough for the welded system to cool to an ambient temperature of 20 °C. Time integration in the calculation step was heat transfer (transient). Development of WRS occurred as the temperature field evolved, the WRS is developed. Lastly, the welded system was cooled to ambient temperature, and thermal analysis was performed. The resulting temperature distribution was regarded as a predefined thermal load performed in the mechanical analysis step.

### 3.2. Mechanical Analysis

In mechanical analysis, as mentioned above, only the boundary condition, meshed element type, and calculation step type were different. Time integration in the mechanical model was generally static. A plane strain bi-linear element with reduced integration element CPE4R was applied for both substrate and cladding. In addition to using the left surface as the symmetry plane, the bottom left corner of the model was fixed. To further control the computational precision, the elements in cladding layer were refined. Element sizes in the cladding layer and substrate were 0.25 × 0.5 mm^2^ and 0.5 × 0.5 mm^2^, respectively. 

During welding, solid-phase transformation was not considered. The total strain rate is composed of three components:

(4) $$\varepsilon =\varepsilon^{e}+\varepsilon^{p}+\varepsilon^{\mathrm{the}}$$

where $\varepsilon^{e}$ is elastic strain, $\varepsilon^{p}$ is plastic strain, $\varepsilon^{\mathrm{the}}$ is thermal strain.

## 4. Results and Discussions

### 4.1. Finite Element Method (FEM) Validation of Elastic Model

Diverse FEMs have been proposed to simulate the WRS in the last few decades, including two and three-dimension, single and multi-pass FEM. However, these FEMs have rarely been compared to analytical models. In this study, prior to elastic–plastic analysis, the elastic analysis model was first established and compared to the analytical model for verification. Unlike the elastic–plastic model, the yield stresses of 2.25Cr–1Mo–0.25V and 347L steel type were not applied in the elastic model. Other parameters in Figure 5 and Figure 6 were not changed.

**Figure 7:**
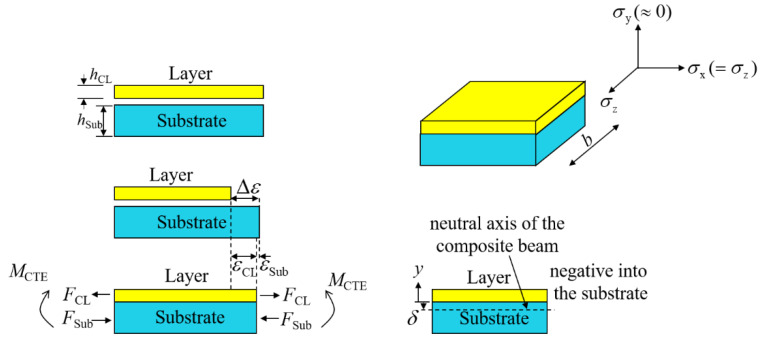
Schematic depiction of generation of pair of equal and opposite forces, and unbalanced moment due to deposition of some of the cladding layer on substrate surface.

The fabrication mechanism of thermal barrier coatings (TBCs) is similar to the cladding process; both deposit the molten metal on the surface of a substrate, as shown in Figure 7. Residual stress generated in TBC fabrication has been widely studied. According to Y. Song and Y.C. Tsu’s studies [34,35,36], WRS generated in the single-layer cladding and substrate after the deposition of cladding layer in this study is shown as follows.

The misfit strain is:

(5) $$\Delta \varepsilon =\frac{\sigma_{q}}{E_{\mathrm{CL}}^{*}}$$

where $\sigma_{q}$ is quenching stress, and $E_{\mathrm{CL}}$ is the Young’s modulus of cladding layer. A pair of equal and opposite forces, *F*_CL_ was set up by misfit strain. The strain equation is:

(6) $$\Delta \varepsilon =\varepsilon_{\mathrm{CL}}-\varepsilon_{\mathrm{Sub}}=\frac{F_{\mathrm{CL}}}{bh_{\mathrm{CL}}E_{\mathrm{CL}}^{*}}+\frac{F_{\mathrm{Sub}}}{bh_{\mathrm{Sub}}E_{\mathrm{Sub}}^{*}}$$

The pair of equal and opposite forces shown in Figure 7 generated a bending moment *M*^CTE^ given by:

(7) $$M_{}^{\mathrm{CTE}}=F_{\mathrm{CL}}^{}\left(\frac{h_{\mathrm{CL}}+h_{\mathrm{Sub}}}{2}\right)$$

The neutral axis position *δ* can be expressed as follows:

(8) $$\delta =\frac{1}{2}\frac{E_{\mathrm{CL}}^{*}h_{\mathrm{CL}}{}^{2}-E_{\mathrm{Sub}}^{*}h_{\mathrm{Sub}}{}^{2}}{E_{\mathrm{CL}}^{*}h_{\mathrm{CL}}+E_{\mathrm{Sub}}^{*}h_{\mathrm{Sub}}}$$

The composite beam stiffness, *D* can be expressed as follows:

(9) $$D=\frac{b}{3}E_{\mathrm{CL}}^{*}\left[{\left(h_{\mathrm{CL}}-\delta \right)}_{}^{3}-{\left(0-\delta \right)}_{}^{3}\right]+\frac{b}{3}E_{\mathrm{Sub}}^{*}\left[{\left(0-\delta \right)}_{}^{3}-{\left(-h_{\mathrm{Sub}}-\delta \right)}_{}^{3}\right]$$

Balancing the moment, *M*^CTE^ induced a curvature change Δ*Κ*^CTE^ that was equal to:

(10) $$\Delta \kappa_{}^{\mathrm{CTE}}=\frac{M^{\mathrm{CTE}}}{D}=\frac{F_{\mathrm{CL}}^{}\left(\frac{h_{\mathrm{CL}}+h_{\mathrm{Sub}}}{2}\right)}{\frac{b}{3}E_{\mathrm{CL}}^{*}\left[{\left(h_{\mathrm{CL}}-\delta \right)}_{}^{3}-{\left(0-\delta \right)}_{}^{3}\right]+\frac{b}{3}E_{\mathrm{Sub}}^{*}\left[{\left(0-\delta \right)}_{}^{3}-{\left(-h_{\mathrm{Sub}}-\delta \right)}_{}^{3}\right]}$$

The Young’s modulus in the above equations had the effective Young’s modulus value E*, expressed as follows:

(11) $$E_{}^{*}=\frac{E}{1-\nu }$$

In this analytical solution, only the elastic strain is considered; WRS distribution through substrate thickness was linear. Therefore, only WRS at the top and bottom of the substrate had to be calculated, as follows:

(12) $$\sigma_{\mathrm{Sub}}|{}_{y=-h_{\mathrm{Sub}}}=\frac{-F_{\mathrm{CL}}}{bh_{\mathrm{Sub}}}+E_{\mathrm{Sub}}\Delta \kappa_{}^{\mathrm{CTE}}\left(h_{\mathrm{Sub}}+\delta \right)$$

(13) $$\sigma_{\mathrm{Sub}}|{}_{y=0}=\frac{-F_{\mathrm{CL}}}{bh_{\mathrm{Sub}}}+E_{\mathrm{Sub}}\Delta \kappa_{}^{\mathrm{CTE}}\delta$$

WRS at the cladding-layer midpoint was calculated from:

(14) σCL|y=hCL2=FCLbhCL−ECLΔκCTE(hCL2−δ)

**Figure 8:**
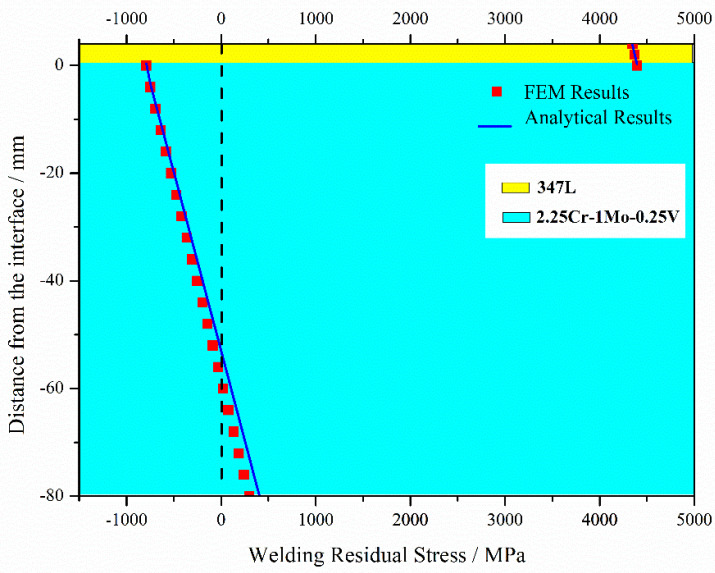
Welding residual stress (WRS) calculated from analytical and numerical simulations of the elastic model.

Figure 8 shows the results of the analytical solutions and numerical simulations of the elastic model when ignoring material plasticity. The WRS tendency indicated by the FEM fits perfectly with that of the analytical solutions. In the cladding layer, WRS is tensile, and stress at the near-surface of the cladding is smaller than that of the interface. The stress in the substrate is partly tensile and partly compressive. The peak WRS magnitude of is 4000 MPa in the cladding layer and −800 MPa in the substrate, significantly higher than the yield stress in practice. Therefore, plasticity cannot be ignored in the numerical simulation of WRS.

### 4.2. Experimental Results

**Figure 9:**
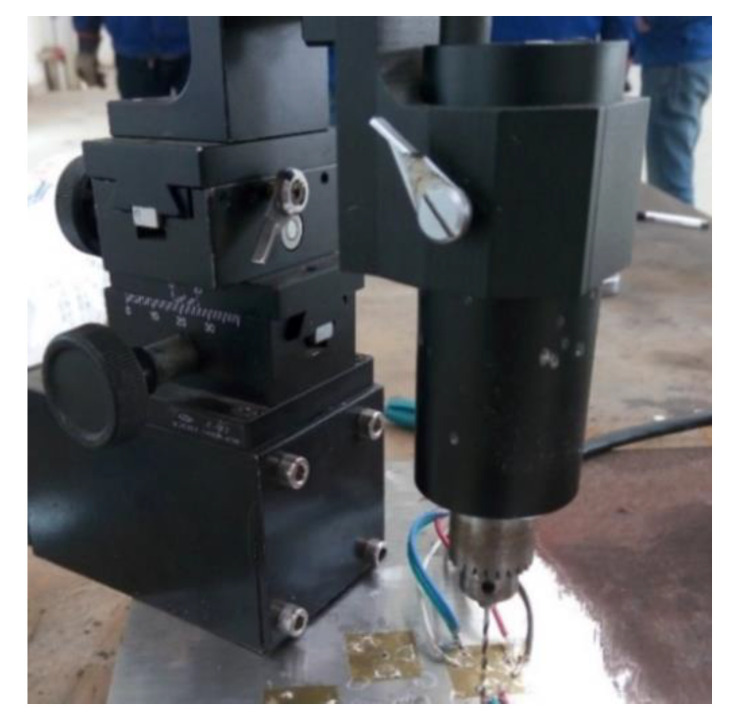
High-speed drilling machine HTZ–12S.

A blind hole method was implemented on the top and bottom surface of the welded specimen. The high-speed drilling machine HTZ–12S was chosen to be the drilling device, as shown in Figure 9. The main technical parameters of this device are as follows: aim-mid precision: ±0.001, hole depth control precision: ±0.05, maximum speed: 4000 r/min and bit clamping range: 0.3–4 mm. The resistance and sensitivity coefficient of strain gauge BHI 120-3CD (11) were 119.5–121.0 ± 0.5 Ω and 2.09% ± 1%, respectively. The depth and diameter of the blind hole were 2 mm and 1.5 mm, respectively. B-702 room temperature curing patch adhesive from AVIC Electrical Measuring Instruments Co. (Hanzhong, Shaanxi, China). was selected for sticking strain gauges. The characteristics of this patch adhesive are strong adhesion, low creep and hysteresis, poor temperature and humidity resistance. It is suitable for the adhesive of room temperature strain and stress test with short cycle. The strain measurement instrument was NI-9235 from National Instruments Co., Ltd (Austin, TX, USA). It is characterized by high accuracy (0.1%), high cost performance, flexibility and ease of use.

**Figure 10:**
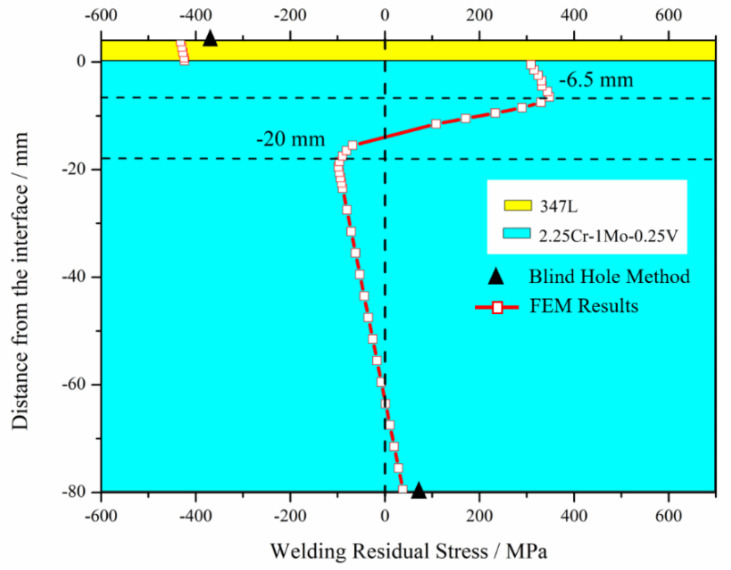
Final WRS distribution of elastic–plastic model and comparison with blind hole method.

The plasticity of the 347L and 2.25Cr–1Mo–0.25V steel types was assigned to the model on the basis of the elastic model, temperature-dependent plasticity is shown in Figure 5 and Figure 6. The final WRS distribution of the elastic–plastic model and comparison with the experimental results are shown in Figure 10. It can be seen that the experimental value on the top surface of the cladding layer is −377.58 MPa (The negative indicates that the WRS is compressive), which is smaller than the absolute value of the FEM value −432.93 MPa. The relative error between the FEM value and the experimental value is 14.66%. WRS on the bottom of the substrate is 75.98 MPa tensile stress. This result is obviously larger than the FEM result (39.08 MPa). The error of the FEM value relative to the experimental value reaches 48.57%. The reason why the bottom surface has such a large error may be that the deformation of the 4 passes cladding layer in the experiment is greater than that of the simplified single pass cladding layer in the FEM. In summary, for residual stress measurement, the results of the FEM were in good agreement with the experimental values. Therefore, the experimental results verified directly the accuracy of the elastic-plastic model.

Moreover, from Figure 10, the following observations were made: (i) WRS distribution could be divided into three parts―cladding layer (0 mm–4 mm, from welding interface to top surface of the cladding layer), SAL (–20 mm–0 mm, from the top part of the substrate adjacent to welding interface), and substrate (from –80 mm to –20 mm); (ii) stress in the cladding layer was compressive [10]; (iii) stress in the SAL was partly tensile and partly compressive; and (iv) stress in the substrate was partly tensile and partly compressive.

### 4.3. Welding Residual Stress (WRS) Evolution of Elastic–Plastic Model

**Figure 11:**
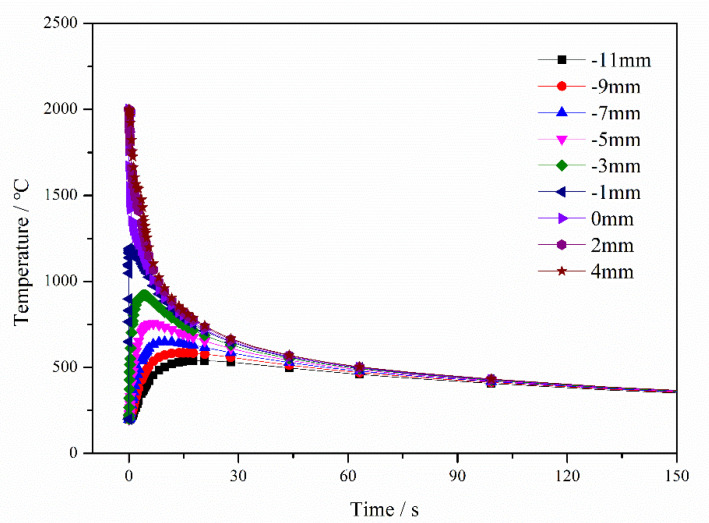
Temperature histories of elements in cooling (values from −11 to 4mm represent the element distance from the interface, and elements at cladding layer expressed as positive; cladding layer (4, 2 and 0 mm), SA L(−1, −3, −5, −7, and −9 mm) and substrate (−11 mm)).

WRS evolution in these three parts is discussed in detail. The element at 1.75 mm in the cladding layer was selected as representative for the entire cladding layer. Figure 11 shows the first half of the cooling process of the element at 1.75 mm. According to the behavior of plastic strain, the cooling process of the cladding layer could be divided into three stages: (i) 0–2.5 s, where 347L steel becomes tensile yielding at 2.5 s; (ii) 2.5–154 s, where plastic strain reaches a maximum at 154 s; and (iii) 154–4000 s, where plastic strain maintains its maximal value.

**Figure 12:**
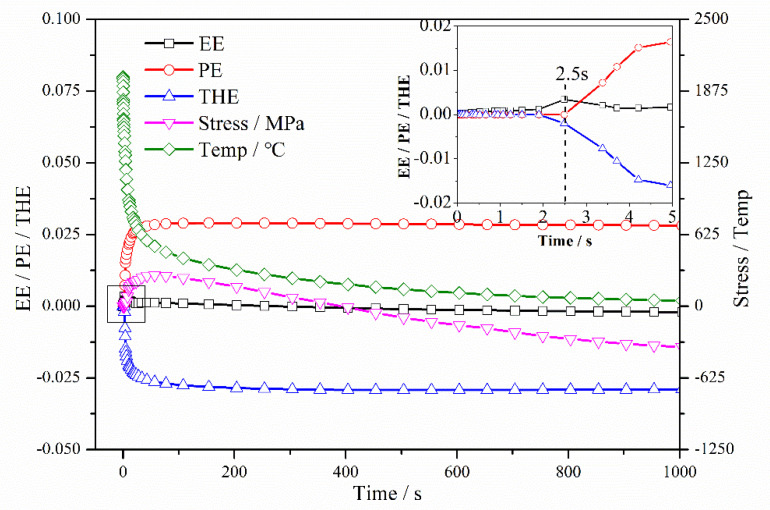
Temperature, strain, and WRS evolution in first half of cooling for elements in cladding layer at 1.75 mm (EE, elastic strain; PE, plastic strain; THE, thermal strain; Stress, WRS; Temp, temperature).

**Figure 13:**
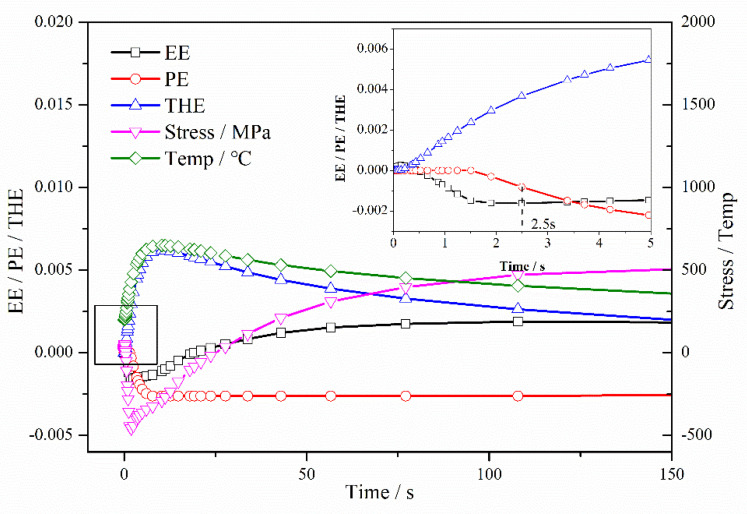
Temperature, strain, and WRS evolution in first half of cooling for element in cladding layer at −6.5 mm (EE, elastic strain; PE, plastic strain; THE, thermal strain; Stress, WRS; Temp, temperature).

In the first stage, the cladding layer was cooled to a solid, and it generated contractive thermal strain, as shown in Figure 11. The temperature trend of the SAL is consistent with the trend of the substrate, which was different from the cladding layer. The temperature of the SAL and the substrate first rapidly increased and then slowly decreased with the progression of welding. The initial temperature of the cladding layer was about 2000 °C and it slowly decreased due to exposure to 20 °C 2.25Cr–1Mo–0.25V steel. The increased temperature of the zone adjacent to the interface caused a corresponding increase in the thermal strain of the SAL (300 °C at –10.5 mm and 1130 °C at –0.5 mm). The temperature histories of elements in the cladding and SAL are shown in Figure 12. Hence, under the influence of contractive thermal strain of the cladding layer and the increase in thermal strain in the SAL (from –20 mm to 0 mm), the element in the cladding layer began to yield at 2.5 s. The SAL, represented by elements at –6.5 mm in the first stage adjacent to the interface, began to compress. At the end of Stage 1, the plastic strain at –6.5 mm was –8.14 × 10^−4^, as shown in Figure 13. In this stage, there was no obvious change in substrate temperature and strain.

In the second stage, the cladding layer continued to cool to ~362 °C, and the temperature of the entire SAL was maintained at ~350 °C. At the end of this stage, the plastic strain of the cladding layer reached a maximal value of 2.90 × 10^−2^, as shown in Figure 12. The plastic strain of the element at −6.5 mm in SAL reached a maximum value of −2.62 × 10^−3^, as shown in Figure 13. At the end of this stage, no significant differences were observed in the temperature distributions between cladding, SAL, and substrate, as shown in Figure 11.

Therefore, the third stage was a relatively gentle process for changes in temperature, strain, and WRS, i.e., the final states of strain and WRS were fundamentally shaped at the end of the second stage. With the combined effect of plastic strain (cladding layer (2.90 × 10^−2^), SAL (from 0 to −2.55 × 10^−3^)), thermal strain (cladding layer (−2.81 × 10^−2^), SAL (from 1.57 × 10^−2^ to 2.13 × 10^−2^)) and elastic strain (cladding layer (6.38 × 10^−4^), the SAL (from −7.69 × 10^−4^ to 1.82 × 10^−3^)), the WRS in the cladding layer was tensile.

In the third stage with uniform cooling, there was a gradual effect of the plastic strain that was generated in the two former stages. The elongated plastic strain of the cladding layer led to compressive WRS. By contrast, the constringent plastic strain of the SAL led to tensile WRS, as shown in Figure 10.

**Figure 14:**
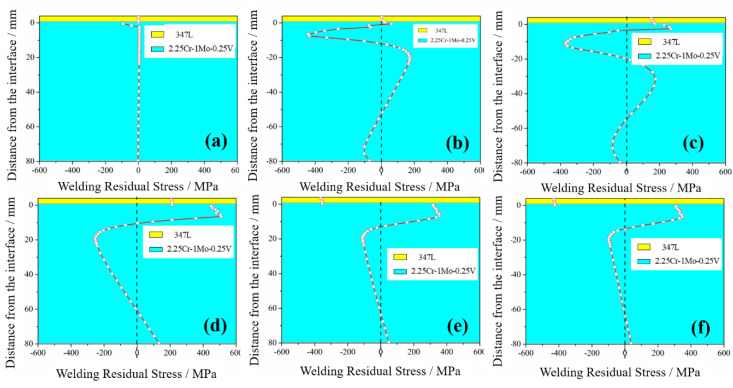
WRS distribution in cladding/substrate system for different cooling times: (**a**) 0.001 s, (**b**) 2.5 s, (**c**) 11.24 s, (**d**) 154 s, (**e**) 1004 s, and (**f**) 4000 s.

**Figure 15:**
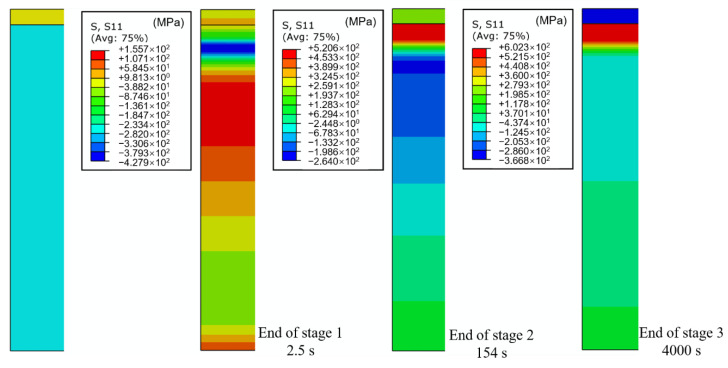
WRS nephogram at the ends of different stages.

Figure 14 shows the evolution of WRS with cooling time. Figure 15 shows WRS nephograms for the different stages. In accordance with the above discussion, the cooling process could be divided into three stages. In the first stage at 0–2.5 s, the dramatic change in temperature led to unstable WRS distribution. However, WRS values remained small except for in the SAL. In the second stage, WRS was fundamentally shaped, except for in the cladding layer. As mentioned above, at the end of the second stage, the plastic strain of the cladding layer and WRS reached the maximal value. In the third stage, there was a gradual change in WRS of entire substrate. The cladding layer was compressed by uniform cooling in the third stage as a result of elongated plastic strain in the cladding layer and constringent plastic strain in the SAL.

### 4.4. Effect of Substrate Thickness

**Figure 16:**
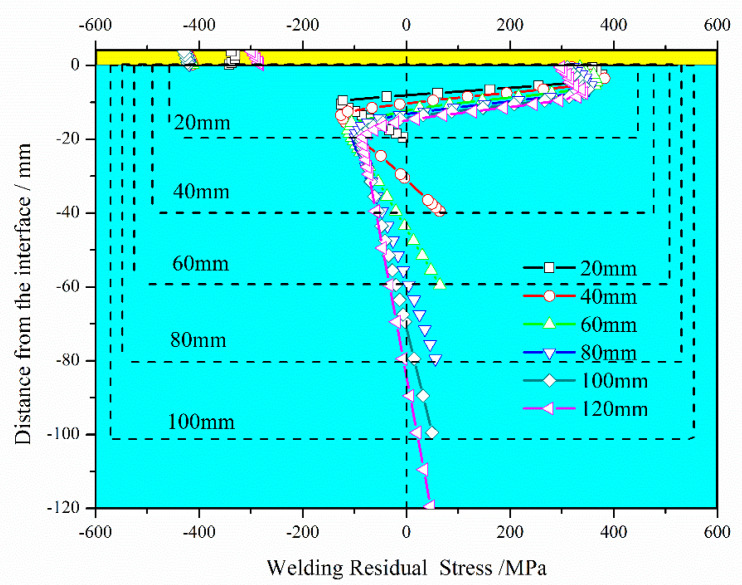
Final WRS for different thicknesses of 2.25Cr–1Mo–0.25V steel substrate.

The substrate is usually required to have different thicknesses for different working conditions. The thickness of the 347L cladding layer and 2.25Cr–1Mo–0.25V substrate significantly affected WRS distribution. However, under different working conditions, the thicknesses of single-layer ESSC were the same. In this study, only the effects of substrate thickness were evaluated, as shown in Figure 16.

The following observations can be made from Figure 16. (i) The tendency of final WRS was the same regardless of substrate thickness (WRS could be divided into three parts: cladding layer, SAL, and substrate). (ii) When substrate thickness was small (<40 mm in this study), both maximal and minimal WRS in the SAL increased with the increase in substrate thickness, as shown in Table 2. When the substrate was sufficiency thick (≥40 mm), both maximal and minimal WRS in the SAL decreased with the increase in substrate thickness. (iii) Final WRS distributions in the SAL and substrate thickness were not significantly affected, but SAL thickness gradually increased from 10.5 mm to 19.5 mm as substrate thickness increased.

### 4.5. Effect of Pre-Heat Temperature

**Figure 17:**
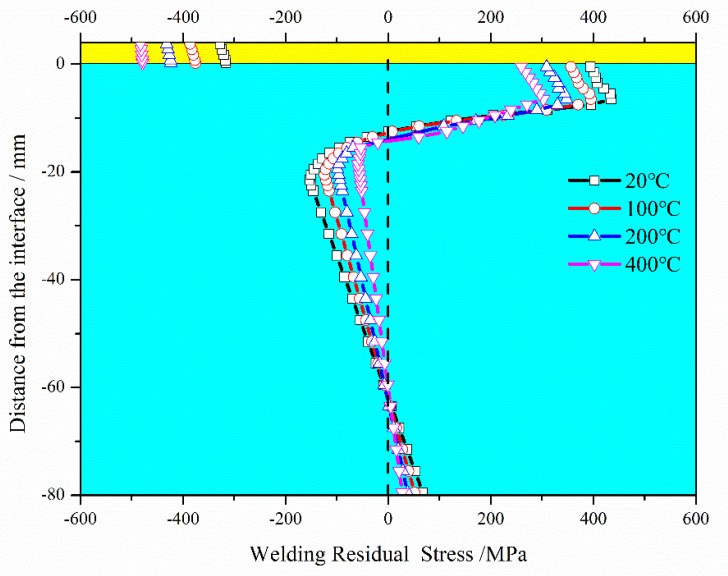
Final WRS with different substrate preheat temperatures levels.

Preheat temperature significantly affected welding quality and service performance, and especially weldability, WRS, and weldment toughness. Figure 17 shows the effect of different preheat temperature levels (20 °C, 100 °C, 200 °C and 400 °C) in terms of final WRS state. The following observations were made: (i) by improving preheat temperature, the thermal mismatch stress between the cladding layer and substrate decreased, leading to a decrease in the substrate WRS. (ii) In the SAL, the final WRS decreased as the preheat temperature was increased to 400 °C. SAL thickness decreased from 21.5 mm without preheat to 18.5 mm with 400 °C preheat. (iii) Compressive WRS in the cladding layer, on the other hand, increased with the increase in preheat temperature.

## 5. Conclusions

In this study, an FEM was proposed by considering yield strength in a cladding/substrate system during the ESSC process to analyze WRS evolution. The effects of welding parameters (preheat temperature) and geometric factors (substrate thickness) were also evaluated. The following conclusions were obtained:

1. WRS distribution could be divided into three regions (cladding layer, SAL, and substrate) as distinct from other studies. In the traditional 200 °C preheat temperature level, WRS in the cladding layer is compressive, and WRS in the SAL and substrate is partly tensile and partly compressive.
2. WRS evolution could be divided into three stages according to the plastic strain state of the materials. Residual stress was rapidly generated within 2.5 s, reaching about −440 MPa compression stress in the SAL region. After cooling for 154 s, WRS was fundamentally shaped, except for in the cladding layer.
3. Substrate thickness significantly affected final WRS. Final WRS in the cladding layer initially increased with substrate thickness. However, WRS started to decrease when the substrate was sufficiently thick. WRS in the SAL increased with substrate thickness until residual stress reached yield stress.
4. The magnitude of WRS in the substrate and SAL decreased with the increase in substrate preheat temperature. By contrast, the compressive WRS in the cladding layer increased with the increase in preheat temperature.

## Figures and Tables

**Table 1:** Chemical composition (wt.%) and heat treatment conditions.

Element	C	S	P	Si	Mn	Cr	Ni	Mo	Cu	V
Base material	0.15	0.01	0.009	0.1	0.54	2.3	0.05	0.98	0.02	0.3
Normalizing Tempering	910 °C/Furnace cooling 720 °C/Air cooling									

**Table 2:** Results for different substrate thicknesses. Note: SAL, stress-affected layer.

Thickness (mm)	WRS in the Cladding Middle (MPa)	Max. WRS in SAL (MPa)	Min. WRS in SAL (MPa)	WRS in Substrate Bottom (MPa)	SAL Thickness (mm)
20	−330.80	377.36	−125.45	6.66	10.5
40	−416.09	382.00	−126.50	64.18	13.5
60	−416.49	366.749	−109.83	64.52	18.5
80	−422.34	355.61	−98.30	55.96	19.5
100	−424.28	350.06	−89.81	49.37	19.5
120	−289.09	342.284	−83.72	46.71	19.5
